# F8. SEARCHING FOR A STRATIFICATION MARKER FOR ANTIOXIDANT USE IN SCHIZOPHRENIA AND BIPOLAR DISORDER: A META-ANALYSIS OF MRS STUDIES OF ANTERIOR CINGULATE GLUTATHIONE

**DOI:** 10.1093/schbul/sby017.539

**Published:** 2018-04-01

**Authors:** Avyarthana Dey, Alborz Javadzadeh, Priyadharshini Sabesan, Joaquim Radua, Jean Theberge, Lena Palaniyappan

**Affiliations:** 1 University of Western Ontario; 2 London Health Sciences Center; 3 King’s College London; 4 St. Joseph’s Health Care London

## Abstract

**Background:**

Glutathione [GSH] is a major intracellular antioxidant that disposes peroxides and protects neurons and glial cells from oxidative stress. In both schizophrenia and bipolar disorder, atypical levels of GSH has been demonstrated, particularly in the anterior cingulate cortex (ACC), though no consistent results have emerged due to limitations in sample size. Examining the state of GSH deficit in schizophrenia is a critical step when attempting to correct putative redox imbalance in this illness using agents such as N-Acetyl Cysteine (NAC). We conducted a meta-analysis to investigate the aberrations in GSH levels in the ACC of patients with schizophrenia and bipolar disorder measured using magnetic resonance spectroscopy (MRS).

**Methods:**

Medline, Google Scholar, Ovid Online and EMBASE databases were searched for studies published until September 2017. Search terms included magnetic resonance spectroscopy, MRS, schizophrenia, psychosis, psychotic, bipolar disorder, glutathione, GSH. We included all 1H-MRS studies reporting GSH values for patients satisfying DSM or ICD based criteria for a primary psychotic disorder (SCZ) or bipolar disorder (BPAD) in comparison to a healthy controls (HC) group. We screened all identified abstracts, filtered studies that did not satisfy inclusion criteria, hand-searched references and contacted experts to locate further studies. We excluded studies that reported only on comorbid illnesses, did not compare patients and HCs, or failed to report data required to construct effect size metrics. After initial screening, a total of 261 patients and 185 controls were considered for the meta-analysis from the SCZ group; 464 patients and 245 controls were considered for the meta-analysis from the BPAD group. A random-effects, inverse-weighted variance model was used to calculate the pooled effect size. Mean values were extracted and verified independently. Effect sizes were computed based on excel macro, produced by Major Depressive Disorder Neuroimaging Database investigators.

**Results:**

Contrary to our expectations, in SCZ, there were no significant differences in ACC GSH in patients compared to HC (RFX p= 0.74; 95% CI, -0.24 to 0.17; FFX p= 0.71; heterogeneity p = 0.58). In BPAD, there were highly significant differences in the ACC GSH, with patients having higher GSH concentrations than HC (RFX: p= 0.0003; 95% CI, 0.14 to 0.5; heterogeneity p=0.70). In the BPAD group, the mean effect size (SMD) was d= 0.32, indicating a small to medium sized difference. A network meta-analysis revealed significantly higher GSH levels in BPAD compared to SCZ (RFX p= 0.01; 95% CI, 0.08 to 0.63; SMD=0.36; heterogeneity p = 0.71). There were several methodological issues in the reported studies. Notably, most acquisitions were not optimized to collect GSH spectra; polymorphisms in the glutamate-cysteine ligase catalytic gene (GCLC) were not quantified in most studies; voxel placements were not standardized across the published studies; majority of patients were medicated, in various stages of illness.

**Discussion:**

There are no major differences in concentration of ACC glutathione in the anterior cingulate cortex in patients with schizophrenia, though in bipolar disorder, GSH levels appear elevated. Given that GSH is the most readily accessible cortical redox marker in vivo, current status of MRS literature is insufficient to prepare for stratified therapeutics with antioxidants among patients with schizophrenia. Nevertheless, abnormalities in the redox system may be more pronounced in bipolar disorder compared to schizophrenia, and could serve to guide stratification in samples lacking diagnostic clarity (e.g. in First Episode Psychosis clinics).

